# Why Do Dolphins Carry Sponges?

**DOI:** 10.1371/journal.pone.0003868

**Published:** 2008-12-10

**Authors:** Janet Mann, Brooke L. Sargeant, Jana J. Watson-Capps, Quincy A. Gibson, Michael R. Heithaus, Richard C. Connor, Eric Patterson

**Affiliations:** 1 Department of Biology, Georgetown University, Washington D. C., United States of America; 2 Department of Psychology, Georgetown University, Washington D. C., United States of America; 3 Department of Biological Sciences, Florida International University, Miami, Florida, United States of America; 4 Department of Biology, Metropolitan State College of Denver, Denver, Colorado, United States of America; 5 Department of Biological Sciences, Marine Sciences Program, Florida International University, North Miami, Florida, United States of America; 6 Department of Biology, University of Massachusetts at Dartmouth, Dartmouth, Massachusetts, United States of America; The University of New South Wales, Australia

## Abstract

Tool use is rare in wild animals, but of widespread interest because of its relationship to animal cognition, social learning and culture. Despite such attention, quantifying the costs and benefits of tool use has been difficult, largely because *if* tool use occurs, all population members typically exhibit the behavior. In Shark Bay, Australia, only a subset of the bottlenose dolphin population uses marine sponges as tools, providing an opportunity to assess both proximate and ultimate costs and benefits and document patterns of transmission. We compared sponge-carrying (sponger) females to non-sponge-carrying (non-sponger) females and show that spongers were more solitary, spent more time in deep water channel habitats, dived for longer durations, and devoted more time to foraging than non-spongers; and, even with these potential proximate costs, calving success of sponger females was not significantly different from non-spongers. We also show a clear female-bias in the ontogeny of sponging. With a solitary lifestyle, specialization, and high foraging demands, spongers used tools more than any non-human animal. We suggest that the ecological, social, and developmental mechanisms involved likely (1) help explain the high intrapopulation variation in female behaviour, (2) indicate tradeoffs (e.g., time allocation) between ecological and social factors and, (3) constrain the spread of this innovation to primarily vertical transmission.

## Introduction

**Figure 1:**
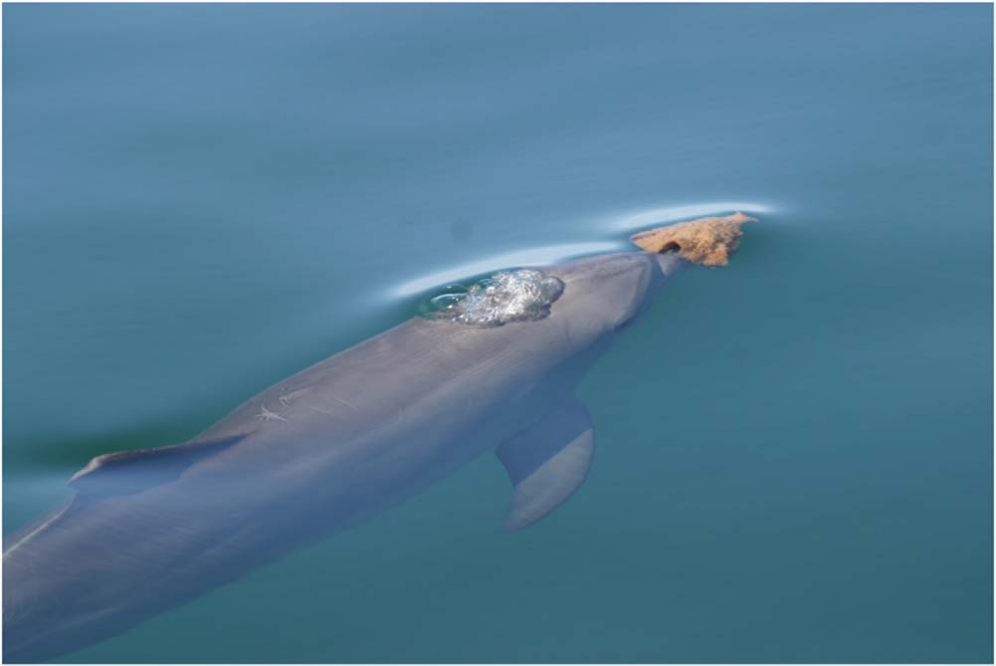
Photograph of Sponger. Courtesy of Ewa Krzyszczyk.

Tool use [1], [2] is rare in the wild, documented in 0.01% of non-primate mammalian species [3], 10 primate species [4], [5] and 30 bird species [6]. In wild animal populations, the adaptive function of tool use behaviour is posited or assumed, but rarely tested, either because tracking the spread of behaviours is challenging or all individuals in a population engage in the tool using behaviour. However, in one of the only suspected cases of tool use for any wild dolphin or whale, sponge-carrying (hereafter sponging) in Shark Bay, Australia [7], only 11% of adult female bottlenose dolphins (*Tursiops* sp.) carry marine sponges [8] (Fig. 1). Tool use in this population is striking compared to tool use in other species because of the degree of specialization, strong sex-bias, and matrilineal (vertical) transmission within a subset of the population [8], [9]. In addition, with the exception of chimpanzees [10] and humans, habitual tool use to hunt vertebrates has not been documented. To date, however, sponging, which occurs almost exclusively in deep water channels [11] has been difficult to observe, and consequently has not been described in detail. As a result, how and why dolphins sponge and whether the behavior is cultural is under debate [e.g.], [ 12]–[14]. In this study, we (1) documented and described the sponging behaviour in detail; (2) described the full sub-population of spongers in our study area; (3) compared sponging to other deep water foraging methods; and (4) determined the costs and benefits of sponging by contrasting the sociality, time budgets, habitat use patterns and calving success of sponging females with other females in the population. By comparing dolphins that use sponges to those that do not, we examined whether the behaviour is advantageous, making the “best of a bad situation” (i.e., when subordinate or less-competitive individuals reduce competition by adopting an alternative tactic at some fitness cost) [15], or has no apparent benefit over other tactics. In the latter case, sponging might be at equilibrium with other foraging tactics, providing no net benefit or cost relative to other techniques [16]. In sum, we are the first to examine the relationship between tool use and fitness in wild animals.

## Results

During foraging, sponge-carrying dolphins wore conical marine sponges (10–25 cm from base to top) over the rostrum, cupping the jaw completely (Fig. 1). Long dives with multiple breaths at the surface were interspersed with rapid single breaths or leaps, typically without the sponge, when prey chases appeared to be underway. The behaviour was highly stereotyped and solitary. For 10 focal sponger females, we examined diving and surfacing behaviour during sponging (i.e., a sponge was worn at least once during each foraging bout). We could determine if females carried sponges on 1,295 surfacing bouts (period at surface between dives). Females surfaced with sponges after 77.3±2.0% of all dives. Females were more likely to surface without sponges during brief surfacings than during longer surfacings (Paired t-test, T = 5.43, P = 0.0004). During long surfacings (>3 sec), females wore sponges 80.3±2.2% of the time, but wore sponges only 24.5±5.9% of brief surfacings (<3 sec at surface; 10.3% of all surfacing bouts). Thus, sponges were apparently used while searching and typically not while chasing prey. Since the sponge cups over the rostra, it cannot be worn during prey capture. In 71 min of detailed observations of four foraging sponge-carriers (on separate days) when water clarity was exceptional, individuals swam slowly along sand-bottom habitats with a sponge on, slightly and intermittently disturbing the seafloor. When prey were apparently detected, the dolphins dropped the sponge, accelerated about 5–10 m and then probed the seafloor with their beaks. Occasionally rapid single breaths or leaps without the sponge were observed before returning to the same spot, indicating that prey may burrow in the sand. Subsequently, the dolphins retrieved sponges and began the search process again. Spongers occasionally surfaced with small fish (<20 cm) that were rapidly swallowed whole. Field observations, photographs and sponge-carrying by human divers (with a sponge cupped over one hand), revealed that the prey were probably small bottom-dwelling fish (e.g., *Parapercis* sp.). During four hours of human sponging, the same fish species, spothead grubfish, *Parapercis clathrata*, was repeatedly ferreted from the seafloor in an area where two dolphins were sponging hours before. The fish were invisible to divers until disturbed by the sponge, at which point they quickly moved several meters away and began to burrow again in the sand. The fish were thus briefly visible and could be readily located even after burrowing. A blurry photo of a female sponger with a fish in her mouth is consistent with our observations of *P. clathrata*. Finally, *P. clathrata* lacks spines and grows to 17 cm, consistent with our finding that they swallow prey quickly and whole. Dolphins searched for up to 10 min for a sponge, transported sponges to foraging areas, and occasionally carried sponges in social groups for later use.

We documented recurrent sponging in 41 individuals: 29 females, 6 males, and 6 of unknown sex. All 41 spongers were observed with sponges during at least 20% of all sightings and/or focal follows, except for one juvenile male who was sighted with a sponge six times out of 95 sighting days. For all 17 cases where maternity was known, the mother was also a sponger (10 daughters, 2 sons, 5 offspring of unknown sex); maternity was not known for the other 24 spongers. Of 33 offspring born to sponger females that survived to weaning, 9 were not observed enough post-weaning (> times) to determine if they carried sponges. Of the 24 remaining, 71% (10 females, 2 males, 5 of unknown sex) became spongers and 29% (one female, 6 males) did not. There were obvious sex differences in the probability of adopting sponging as a foraging tactic and the timing of the development of sponging behavior. Of 19 offspring of *known* sex that were born to spongers and survived to weaning, 91% of the 11 daughters and 25% of the 8 sons adopted sponging (χ^2^ = 7.47 p = 0.006). Furthermore, of offspring born to spongers that were observed in detail during focal follows (216 hrs, 7 females, 5 males), all seven daughters, but no sons, carried sponges *as dependent (nursing) calves*, typically by their second or third year (χ^2^ = 10.40 p = 0.001). Only one of these males was observed with sponges post-weaning. Of 101 offspring born to non-spongers that were also observed during focal follows (2120 hrs), none were observed with sponges.

**Figure 2:**
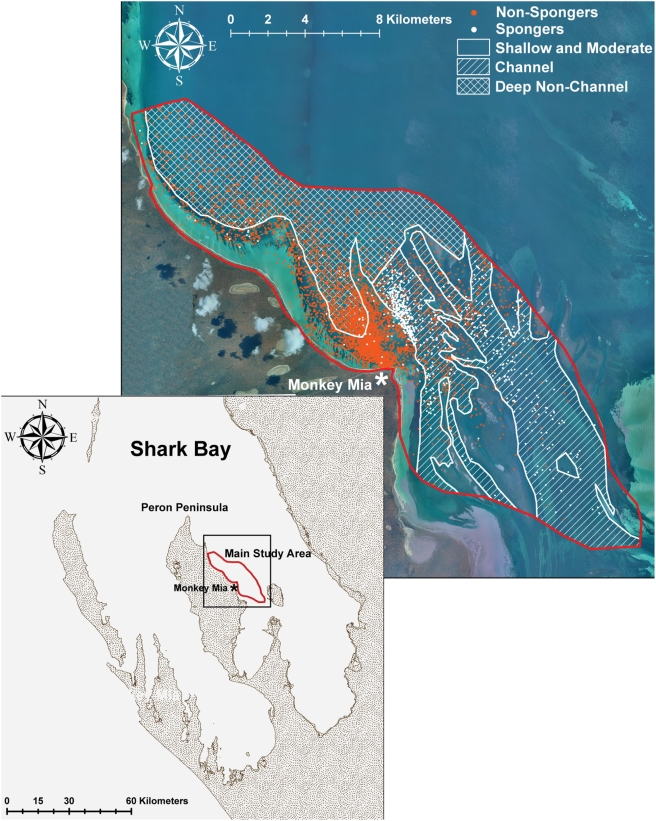
Habitat use of 132 adult female bottlenose dolphins in Shark Bay in 286 km^2^ study area. Image shows sightings of spongers (white circles, N = 16 spongers, 900 sightings, 52.9±12.6 per sponger) with non-spongers (red circles, N = 116 non-spongers, 9,742 sightings, 84.7±11.3 per female) in our main study area using only one sighting per day per female. In both maps, spongers are placed on top of the other females. Three zones are represented: channels (hatched lines), deep water (cross-hatched) and shallow moderate depth (open). Spongers were sighted 84.1±2.8% of the time in channels. Non-spongers were sighted 17.0±2.2% of the time in channels. If the primary area (>50% of sightings) is used to define adult female density by zone, then 32 females use channels as their primary area (female density = 0.34 females per km^2^), including all spongers and 16 non-spongers; 48 females use deep water as their primary area (0.60 females per km^2^) and 44 females use shallow-moderate depths as their primary area (0.40 females per km^2^). Seven females could not be assigned to a primary area. The map shows where spongers were sighted, but sponge foraging only occurred in channel habitats and, on rare occasion, in the deep (>7 m) northwest portion of the study area.

We examined the proximate costs and benefits of sponging by comparing it to 1) the most similar foraging technique, Tail out dive-peduncle dive (TDPD) foraging and 2) a larger sample of 53 focal females with dependent calves. Compared to TDPD foragers, sponge-carriers made more steep descent (tail-out) dives, spent more time in deep water, and were more specialized, spending on average 96% of their foraging time using sponges (Table 1). Sponge-carrying occurred overwhelmingly in deep water channels in the southeastern portion of our study area. Although some TDPD foraging also occurred in these habitats, it was most common in embayment plains outside of the deep water channels (Fig. 2). Although there were no differences in the proportion of time spongers and TDPD foragers spent in foraging bouts, compared to the larger sample of 53 focal females with dependent calves, spongers spent more time foraging (Mean±SE = 53.14±3.34%) than other females (Mean = 29.8±3.0%; T = 4.87; P<0.00001). Spongers also spent a larger proportion of their time alone or alone with their calves (82.69±4.71%) than other females (49.3±4.70%; T = 5.29, p<0.00001). Spongers had fewer associates (15.00±4.80; range = 0–55) overall than other adult females (43.73±6.12, range 0–139; T = 3.66; p = 0.0006).

**Table 1:** Foraging patterns for sponging and TDPD females.

Variable	Sponge-Carrier Mean±SE	TDPD Mean±SE	Z- or t Score	p-value
% of time spent foraging	47.2±4.77	35.84±5.24	−1.59	0.110
Duration of foraging bouts (min)	8.50±1.02	9.59±1.26	−0.75	0.446
Mean dive duration (min)	1.58±0.07	1.64±0.11	−1.00	0.316
% of tail-out dives during foraging	83.47±3.43	65.44±5.17	−2.71	**0.007**
% time in predominant foraging method	45.08±4.91	26.77±5.08	−2.57	**0.010**
% of foraging time devoted to primary foraging type	96.00±2.05	35.84±4.91	−2.41	**0.016**
% of bouts devoted to primary foraging type	93.89±2.65	78.09±5.97	−2.19	**0.028**
% time in deep water when not foraging	93.40±2.44	57.38±7.78	−3.84	**<0.001**

The last five variables in the table show the degree of specialization exhibited by sponger relative to TDPD females.

There were no ultimate costs to sponging in terms of calving success (our best proxy for female fitness). The average calving rate for sponge-carrying females, non-sponge-carrier females and all females was 0.156±0.018, 0.132±0.008 and 0.135±0.007 surviving calves per year, respectively. The model (see methods) without sponge-carrying was slightly better than the model that included sponge-carrying (AIC_c_ = 372.13 vs 368.09) and adequately fit the data (deviance = 100.51; df = 125; p = 0.947), but none of the explanatory variables were significant (all β 95% confidence intervals included zero and all χ^2^ tests based on the reduction in deviance obtained by including a given variable in the model had P≥0.296).

## Discussion

Here we addressed some of the proximate and ultimate mechanisms involved in sponge-carrying, including factors critical to the ontogeny of sponging. Tool use with marine sponges is clearly a foraging behaviour that involves using the sponge to ferret prey from the sea floor. Sponging was strongly female-biased in its occurrence and development and was transmitted vertically. There were no known cases of individuals adopting sponging unless their mothers had also engaged in the behaviour, although genetic data indicate that more than one matriline is involved [9]. Previous research [7]–[9] examined only a small subset of the sponger population in our study area and the description and function of the behaviour were far from complete. Although there are clearly proximate costs to sponging - greater time spent more time foraging at deeper depths, longer dives, and less social interaction, there were no apparent fitness costs, with sponger females having calving success equivalent to non-sponger females.

Females sponged almost exclusively in deep water channels, with some interesting exceptions. Notably, one family of spongers (grandmother, mother, daughter and one unsexed offspring) has been observed sponging periodically in a deep non-channel area in the northwest portion of our study area (see Fig. 2). All but the grandmother were observed as dependent calves accompanying their mothers there, and continued to use this area for sponging post-weaning. They are the only spongers that find and use sponges in this non-channel area, even though it has very few basket sponges compared to the channels [11]. Thus, sponging does not *exclusively* occur in channels, and traditional use of other deep water areas could develop, provided at least some basket sponges are available.

Spongers did, however, constitute at least half the adult female population in deep-water channels. As such, it is the most common female foraging type observed in channel habitats. Perhaps sponging has allowed females to more effectively access prey in channel habitats compared to non-tool users, thus exploiting an otherwise unused niche. That is, employment of sponges allows dolphins to access partially buried prey that would be difficult or costly to find, and/or extract, otherwise. Channels had the lowest adult female density of all major habitat types, suggesting that it is not a superior habitat for all females. Further, it is likely that the sponge-method would not be effective in other habitats (such as dense seagrass) either because the targeted prey are not present, and/or prey capture may not be enhanced by the use of sponges in seagrass habitats. Prey that burrow in sand (as opposed to seagrass) cannot move from that spot and remain hidden. Although targeted prey might be present in other deep water habitats, the density of basket sponges might be too low to support sponging [11]. It is notable that spongers were the only “solitary” females in the channels. Non-spongers that spent more than 50% of their time in channels were alone <30% of the time, in striking contrast to spongers, who were alone >80%. To better contrast the social-foraging profiles of spongers and non-spongers in channel habitats, more observations of the latter group are needed. In addition to habitat, ontogenetic factors clearly determine which females develop sponging.

The sex difference in occurrence and ontogeny of sponging strongly suggests a sex-bias in social learning, similar to the development in termite fishing in chimpanzees [17]. Male offspring are exposed to sponging as often as female offspring, but do not seem to adopt the behaviour early, if at all. This sex-bias may be specific to several foraging types [e.g., 18]. Male offspring otherwise exhibit behaviours similar to their mothers during dependency, although female offspring show stronger similarity to maternal behaviour, a pattern likely driven by sex-specific reproductive strategies [19], [20]. That is, females adopt maternal socio-ecological tactics [19], [20], including habitat use, while males likely range more widely post-weaning, focus on establishing long-term alliances [21], [22], and cannot afford to adopt foraging tactics that both demand extensive effort and specialization and limit their range and access to females. All 6 male spongers are now adults and continue to carry sponges, but whether they specialize in sponging to the degree that females do is not known.

Still, why don't other females adopt the technique? First, offspring, especially daughters, have a stronger tendency to copy their mother's foraging type(s) [8], [18]. With significant habitat heterogeneity, a fission-fusion social structure and female philopatry, the optimal tactic for daughters to adopt is their mothers,' especially if an extensive period of learning is required. Genetic data [9] suggest matrilineal transmission over a longer time scale. If a female's mother is not a sponger, she may have insufficient exposure to develop the technique as a calf. In 83% of 567 surveys where at least one dolphin was wearing a sponge, the sponger was alone or part of a mother-calf pair. In only 6% of sightings (N = 32) was a non-tool user present, and in only 13% of sightings was another sponger present (65 sightings, including 8 where non-spongers were also present). Further, no adult female sponger used sponges for <50% of her foraging budget, indicating a high degree of specialization. Those not born to spongers would have to shift foraging tactics dramatically despite minimal exposure to sponging during early development. We suggest that the identity of the model (mother), sex of observer (female) and frequency of exposure (to maternal foraging and/or channel habitat) are the primary influences on social transmission of sponging.

With extensive overlap in female habitat use and virtually no evidence for female contest competition [23], we argue that spongers are not relegated to channels or preventing others from foraging there. In this case, necessity may not be the “mother of invention”. Clearly, with neutral and potentially positive fitness outcomes, spongers are not making the best of a bad situation. Other studies in diverse systems have documented decreased feeding competition as a result of individual foraging differences [e.g.], [ 24]–[26]. Food-limitation and/or competition and habitat heterogeneity [11] have been proffered as ecological forces that favor individual foraging specialization [27], but such hypotheses are not easily tested with a single population. We did not find differences in calving success among dolphins as a function of habitat use or the use of sponges. However, if fitness of spongers is frequency-dependent, and offspring are not constrained to adopting this tactic, then at equilibrium fitness of spongers and non-spongers should be equal [16].

Behavioural (foraging type, sociality) and ecological (habitat and depth) factors were not predictive of calving success. Given the variation in female calving success, with approximately 17% of females failing to produce surviving calves, factors influencing female reproduction clearly need further attention. The results are consistent with our earlier finding that sociality did not contribute to calving success, but inconsistent with the finding that females in shallow water had higher success than those in deep. There are several possible explanations for this discrepancy. First, we previously used different measures of habitat use and calving success. Second, the current sample included 94 additional females, many more deep water females, and many more reproductive years.

Sponging appears to be an all-or-none phenomenon, requiring a commitment to one foraging type, habitat, and lifestyle. As a result, spongers devote more time to using tools (Table 1) than any non-human species. Although it could be argued that spongers aren't actively using the sponge 100% of the time that they are foraging, if we subtract time they are at the surface (22%) and estimate that they actively used sponges for only half the time they were submerged (during sponge foraging bouts), their “active” tool-use budget would still be more than 17%. Tool use by most non-human species tends to be opportunistic or occasional [3]–[4]. Few studies report tool using time budgets, but even habitual tool users such as orangutans (*Pongo pygmaeus abelii*) [28] and chimpanzees [29] devote a minute portion (<3%) of their overall activity (and foraging) budget to tool use; on the avian extreme is one population of woodpecker finches (*Cactospiza pallida*) that use tools for approximately 10% of the time [calculated from 30]. Likely explanations for this difference are that the primates and birds have more diverse diets, most of which do not require tools. Spongers likely specialize on a small number of fish species, and can only access them readily and reliably with sponges. Another obvious difference is that sponges are used in the search and probably extraction phases of foraging, while primates and birds use tools during the extraction only, which takes less time.

Use of sponges as tools is but one facet of a broader pattern evident in the Shark Bay dolphin population: female dolphins exhibit multiple foraging traditions that are primarily vertically transmitted [also see 8], [11], [18], [31], and are indicative of diverse niche specialization within, rather than between populations or species. Although individual specialization occurs within non-human populations [32], it may be less common in social species. For example, sea otters (*Enhydra lutris*) also show individual foraging specialization [27], but tend to be less social. Social and ecological factors likely favor a prolonged infancy period where extensive exposure to maternal foraging behaviour would promote calf learning and survival post-weaning. Given the impressive array of cognitive skills bottlenose dolphins exhibit in captive studies, such as program-level imitation [33], [34], mental representation [e.g., cross–modal representation of echoic and visual information, 35], exceptional memory [36], and behavioral innovation [37], our findings offer additional insight into those selection pressures that likely favored high individual variation, behavioral plasticity, and a protracted period of social learning and development. These characteristics and niche variation likely fostered the evolution of multiple traditions, including tool-use, in bottlenose dolphins.

## Materials and Methods

### Study Area and Habitat

A long-term study of the dolphins of Shark Bay (25°47′S,113°43′E), Australia, was established in 1984. Using small boats (<5 m), we employ a range of sampling techniques to record individual dolphin behaviour, demography, reproduction, ecology, and genetics. The main study area currently encompasses 286 km^2^ off the east side of the Peron Peninsula (Figure 2) where approximately 550 individual dolphins are being monitored each year. To determine our primary search area, we mapped 5 years (field seasons) of daily GPS tracks totaling 198 days and 71,469 location (latitude and longitude) points. We drew a polygon around the area that encompassed 97% of all points, roughly representing our search effort for the entire study area. The habitat consists mostly of deep embayment plains (6–13 m), shallow sand flats (0.5–5 m), and shallow seagrass beds (0.5–5 m), bisected by deeper channels (6–13 m). We created bathymetry maps (corrected for tidal variation) using 59,234 depth and location points (26,538 from West Australian Department of Planning and Infrastructure transects and 32,696 from our own field research), and sea grass maps from the WA Department of Environment and Conservation. We then divided the study area into three primary zones: (1) shallow and moderate depth (<6 m); (2) deep open habitat (>6 m); and, (3) channels (>6 m) (Figure 2). Shallow and moderate depth (4.23±0.03 m) consists of either sand flats or dense sea grass beds. Deep open habitat (7.50±0.04 m) and channels (8.06±0.04) both have sparse patches of sea grass, but channels have significantly higher basket sponge density than all other habitats [11].

### Surveys and Focal Follows

Surveys included sighting records of individual dolphins or groups [38] lasting a minimum of 5 min and no longer than 1 hr. These represent a “snapshot” of dolphin behaviour, associates, and location. The long-term survey records and focal follows were used to document the occurrence of sponging in juveniles and adults and to determine female calving success. During focal follows, single adult females or mother-calf pairs were followed for 1–9 hr and systematic point sampling and continuous samples were collected on behaviour, diving bouts, mother-calf distance, location, speed, associates and other information [39]. Dive cycles were defined by a series of breaths at the surface, followed by a dive typically lasting 1–3 minutes. Water depth was assessed at 5-min intervals using a depth sounder. We classified sponger females as individuals seen carrying sponges on more than one day in either surveys or focal follows. Tail-out dive/peduncle dive (TDPD) foragers were defined as dolphins that engaged in primarily (>50%) TDPD-foraging bouts. Tail-out dives are steep descent dives where the tail-flukes break the water surface. Peduncle dives occur when the tail-stock (peduncle) is arched at the onset of a dive, but the flukes do not break the water surface. Both sponging and TDPD foraging are the only foraging types that occur primarily in deep water [8]. They are similar in that dolphins make deep water dives averaging 1–2 min and frequently change direction, but TDPD foraging does not include carrying sponges. For the TDPD-sponger comparison, we included 278 focal follows on 26 adult females (727 total hr of observation), of which 14 were spongers (151 hr) and 12 were TDPD foraging females (576 hr). We also compared time budgets for a larger sample of focal females with dependent calves (N = 53 adult females, 13 spongers, 40 non-spongers; 1177 hr) in order to compare all lactating females.

### Female Habitat Use

We used focal follows and survey records to quantify female habitat use. Location (using GPS or compass bearings) was recorded once per survey, and every 5, 15, or 30 min during focal follows. We used only one sighting per female per day to calculate the proportion of sightings each adult female was sighted within and outside of channels and in deep open water (N = 132 females, averaging 81.24±10.03 sightings per female). To reduce sampling bias by search effort (e.g., sightings biased towards boat launch area), we used the last location point per day for each female.

### Calving Success

Female calving success was calculated by dividing the total number of calves surviving to age three (minimum weaning age) by the number of years of known reproductive status (with dependent calf or not). Females were included in this analysis if they were >11 years old and had at least four years of reproductive data. Since calves were rarely weaned before three years [38], the maximum successful calving rate for this sample was 0.29 per year. Calving rate was calculated using the long-term records from the Shark Bay Dolphin Research Project. Calving success of sponge-carriers and TDPD foragers could not be compared directly because our focal data primarily targeted females with dependent calves (i.e., females with some degree of calving success) and females could not be classified as TDPD foragers except by focal observations. Thus, to address whether sponge-carriers had higher calving success than other females, we included our entire longitudinal dataset of 132 females (including 16 sponge-carriers). The number of reproductive years used to calculate female calving success was similar between groups (10.9±.01 [mean±SE] years for all females, 10.9±.02 for spongers and 10.9±.01 for non-spongers).

### Age Estimates

Female age was estimated by known birth year, degree of ventral speckling, first reproductive events, and/or size estimates. Speckling-derived estimates were based on systematic speckle ratings for 63 Shark Bay dolphins of known age (unpublished data). Shark Bay dolphins first begin to speckle in the genital area at age 10, are moderately speckled by 18 and heavily speckled by their late 20 s). Body size assessments and first reproductive event were also used to refine (not define) age estimates. For analysis of calving success, the average age of all adult females was 19.8±0.4 (spongers: 19.5±1.10, non-spongers: 19.8±0.40).

### Statistical Analyses

We used t-tests or Mann-Whitney U tests to compare sponger and non-sponger female focal data. Mean±SE in addition to medians for non-parametric tests are presented. We used generalized linear models [40]) with Poisson distributions and log link functions to evaluate the contributions of sponging (yes or no), water depth (% of sightings in deep water), channel use (% of sightings in channels), age (average age during observation period), sociality (% of surveys with others) to female calving success (# of calves surviving to age three), with the number of years with reproductive data as an offset variable to account for differences in observation effort. We included interaction terms of sponging×channel use and sponging×channel use×sociality. Depth was included in the model because previous research with a much smaller sample indicated a negative relationship with depth use and female calving success [38]. Extensive overlap between sponger and other adult females is evident (Fig. 2), but given that channel use and not sponge-carrying per se might contribute to fitness, we compared a model with sponging included to one without it using AIC_c_ values (a difference ≥2 was considered to show greater support for one of the models; 41). We evaluated absolute model fit of the best model using deviance and evaluated the significance of the parameter estimates using the difference in deviance between the maximal model (model with all explanatory variables included) and the model without a given variable [40].
